# Crystal structure of 4-[(1*R*,2*S*,5*R*)-2-isopropyl-5-methyl­cyclo­hex­yl] 2-methyl (2*S*,4*S*,5*R*)-1-[(2*S*,3*R*,5*R*)-5-meth­oxy­carbonyl-2-(2-methyl­phen­yl)pyrrolidine-3-carbon­yl]-5-(2-methyl­phen­yl)pyrrolidine-2,4-di­carboxyl­ate

**DOI:** 10.1107/S2056989019004079

**Published:** 2019-04-02

**Authors:** Polina M. Ivantcova, Mikhail N. Sokolov, Konstantin V. Kudryavtsev, Andrei V. Churakov

**Affiliations:** a Department of Chemistry, M.V. Lomonosov Moscow State University, Leninskie Gory 1/3, Moscow 119991, Russian Federation; b Institute of General and Inorganic Chemistry, Russian Academy of Sciences, Leninskii prosp. 31, Moscow 119991, Russian Federation

**Keywords:** β-proline oligomers, β-peptides, peptide bond configuration, crystal structure

## Abstract

The title compound, C_38_H_50_N_2_O_7_, represents a chiral β-proline dipeptide. Corresponding stereogenic centres of constituting pyrrolidine units have opposite absolute configurations. The central amide fragment is planar within 0.1 Å and adopts a *Z* configuration along the N—CO bond. In the crystal, the hydrogen atoms of the methyl­ene groups form several short inter­molecular C—H⋯O contacts with the carbonyl oxygen atoms of an adjacent mol­ecule. The only active amino hydrogen atom is not involved in hydrogen bonding.

## Abstract 2

The title compound represents a chiral β-proline dipeptide. Corresponding stereogenic centres of constituting pyrrolidine units have opposite absolute configurations. The central amide fragment is planar within 0.1 Å and adopts a *Z* configuration along the N—CO bond.

## Chemical context   

We have developed an asymmetric protecting-group-free method for the efficient synthesis of alternating β-proline oligopeptides utilizing the stereospecific cyclo­addition of non-racemic homochiral acryl­amides to azomethine ylides (Kudryavtsev *et al.*, 2013 ▸, 2015*b* ▸). Several members of this novel β-peptide class display cell-cycle-directed anti­proliferative activity in hormone-refractory prostate cancer cells (Kudryavtsev *et al.*, 2015*a* ▸,*b* ▸; 2016 ▸). The preference for the *Z* configuration of β-amide bonds in alternating β-proline oligopeptides was explained by inter­action between a lone pair of the carbonyl oxygen atom of the β-amide group and a vacant π* orbital of C^∊^ of the meth­oxy­carbonyl groups (Kudryavtsev *et al.*, 2015*b* ▸).
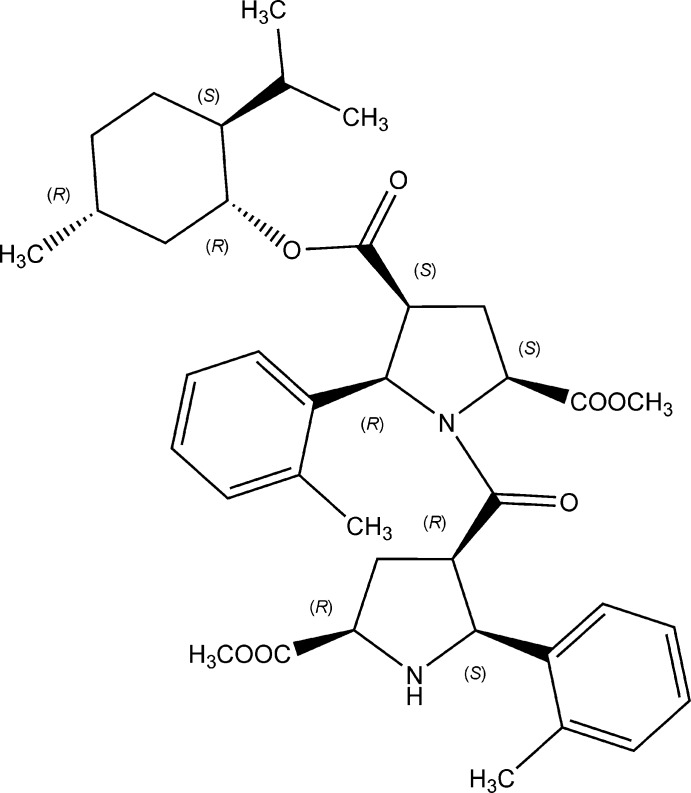


## Structural commentary   

**Figure 1:**
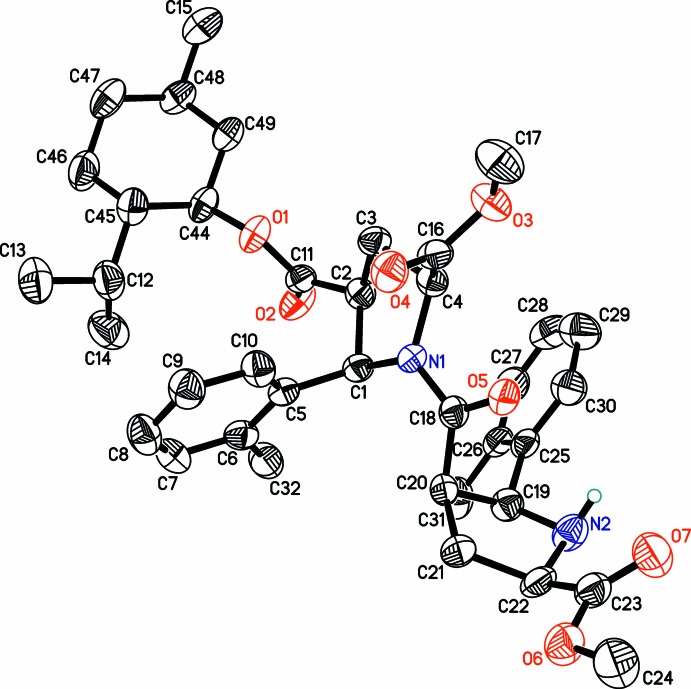
Labelling scheme for the title compound. Displacement ellipsoids are shown at 50% probability level. Hydrogen atoms (except amino H11) were omitted for clarity.

The title compound (Fig. 1 ▸) is a chiral dimeric β-proline derivative. The central amide fragment C4,C1,N1,C18,O5,C20 is planar within 0.1 Å and adopts a *Z* configuration along the N—CO bond. The *Z*/*E* or *trans*/*cis* configuration of a peptide bond is assigned by IUPAC rules due to its partial double-bond character (IUPAC–IUB, 1970 ▸). The amino N2 atom is clearly trigonal–pyramidal with C—N—C and C—N—H bond angles varying from 104.4 (4) to 112 (4)°. Both pyrrolidine rings possess envelope conformations with flap atoms C3 and C19. These atoms deviate from the basal planes of the envelopes by 0.582 (7) and 0.524 Å, respectively. In contrast to the previously reported structures of β-proline oligomers, the flap atoms C3 and C19 are not connected to the amide or carboxyl­ate substituents (see below). Both tolyl groups are almost perpendicular to the pyrrolidine fragments, subtending dihedral angles equal to 84.0 (1) and 75.8 (2)°.

## Supra­molecular features   

**Figure 2:**
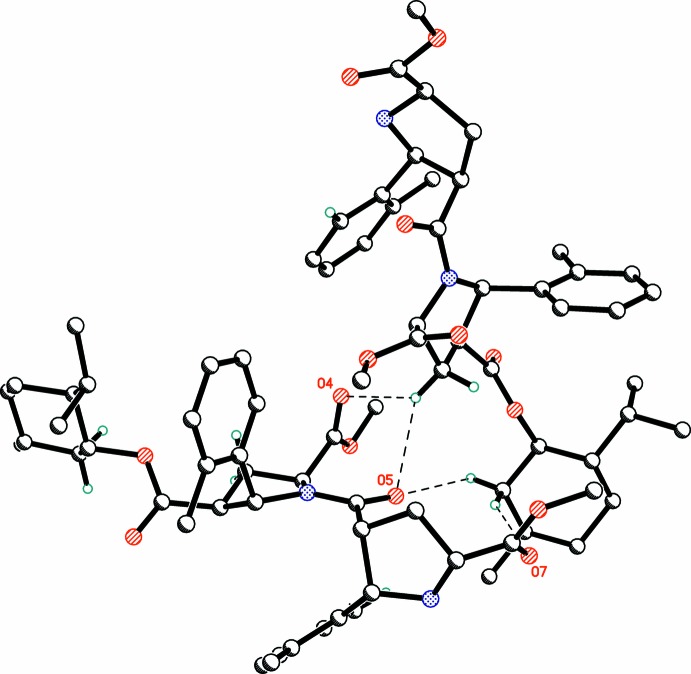
Fragment of the crystal packing showing the shortest inter­molecular C—H⋯O contacts (Table 1 ▸) as dashed lines.

The title mol­ecule contains seven oxygen atoms suitable for hydrogen bonding. Surprisingly, the only active amino hydrogen atom H11 is not involved in hydrogen bonding. This is the result of steric hindrance by the two bulky β-substituents on pyrrolidine ring atom N1. In the crystal, the hydrogen atoms of the methyl­ene groups C3 and C49 form several short inter­molecular C—H⋯O contacts (Table 1 ▸, Fig. 2 ▸) with the carbonyl oxygen atoms O4, O5 and O7 of an adjacent mol­ecule (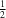 + *x*, 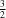 − *y*, 1 − *z*) with H⋯O separations of 2.52, 2.58 and 2.63 Å, respectively. A similar absence of hydrogen bonding has been observed in the structures of closely related β-proline trimers and tetra­mers (Kudryavtsev *et al.*, 2013 ▸, 2015*a* ▸).

## Database survey   

The Cambridge database (version 5.39, Aug 2018; Groom *et al.*, 2016 ▸) contains 11 structures of β-proline oligomers. Among these, three are dimeric (CIKHOV, ILOZOY, and ZUYBUS), three are trimeric [CIKHEL and CIKHIP (Kudryavtsev *et al.*, 2013 ▸) and OWALEF (Kudryavtsev *et al.*, 2016 ▸)] and five are tetra­meric (XOQDOY and XOQDUE (Kudryavtsev *et al.*, 2015*a* ▸), ZUYGUX, ZUYHAE, and ZUYHEI (Kudryavtsev *et al.*, 2015*b* ▸)]. In total, these structures comprises 25 pyrrolidine fragments. Inter­estingly, all 25 pyrrolidine rings adopt envelope conformations with the flap carbon atom bearing linking amide –C(=O)N=or terminal –CO_2_ *R* groups. Endocyclic carbon atoms with aryl substituents and nitro­gen atoms always lie in the basal planes of the proline moieties.

## Synthesis and crystallization   

The synthesis and spectroscopic data for the title compound have been reported by Kudryavtsev *et al.* (2016 ▸). The crystal studied was grown by slow evaporation of a methanol solution of the title compound.

## Refinement   

Crystal data, data collection and structure refinement details are summarized in Table 2 ▸. Aromatic H atoms were placed in calculated positions with C—H = 0.95 Å and refined as riding atoms with *U*_iso_(H) = 1.2*U*_eq_(C). Methyl H atoms were also placed in calculated positions with C—H = 0.98 Å and refined as riding atoms with *U*_iso_(H) = 1.5*U*_eq_(C) and free rotation about the C—Me bonds. The amino H atom was found from the difference-Fourier synthesis and refined with both positional and thermal parameters. As the oxygen atoms are the heaviest in the structure, the absolute configuration could not be determined reliably from the diffraction data. The absolute configuration of the pyrrolidine stereogenic centres was assigned on the base of known chirality of the l-menthol precursor (Kudryavtsev *et al.*, 2016 ▸).

## Supplementary Material

Crystal structure: contains datablock(s) I. DOI: 10.1107/S2056989019004079/eb2015sup1.cif

Structure factors: contains datablock(s) I. DOI: 10.1107/S2056989019004079/eb2015Isup2.hkl

Supporting information file. DOI: 10.1107/S2056989019004079/eb2015Isup3.cml

CCDC reference: 1905705

Additional supporting information:  crystallographic information; 3D view; checkCIF report

## Figures and Tables

**Table 1:** Hydrogen-bond geometry (Å, °)

*D*—H⋯*A*	*D*—H	H⋯*A*	*D*⋯*A*	*D*—H⋯*A*
C3—H3*B*⋯O4^i^	0.99	2.52	3.254 (6)	131
C3—H3*B*⋯O5^i^	0.99	2.58	3.462 (6)	149
C49—H49*A*⋯O7^i^	0.99	2.63	3.511 (7)	149

Symmetry code: (i) 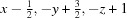.

**Table 2:** Experimental details

Crystal data	
Chemical formula	C_38_H_50_N_2_O_7_
*M*_r_	646.80
Crystal system, space group	Orthorhombic, *P*2_1_2_1_2_1_
Temperature (K)	150
*a*, *b*, *c* (Å)	10.993 (8), 13.198 (10), 23.799 (19)
*V* (Å^3^)	3453 (5)
*Z*	4
Radiation type	Mo *K*α
μ (mm^−1^)	0.09
Crystal size (mm)	0.50 × 0.10 × 0.04

Data collection	
Diffractometer	Bruker SMART APEXII
Absorption correction	Multi-scan (*SADABS*; Bruker, 2008 ▸)
*T*_min_, *T*_max_	0.959, 0.997
No. of measured, independent and observed [*I* > 2σ(*I*)] reflections	19713, 3438, 1990
*R*_int_	0.169
(sin θ/λ)_max_ (Å^−1^)	0.596

Refinement	
*R*[*F* ^2^ > 2σ(*F* ^2^)], *wR*(*F* ^2^), *S*	0.060, 0.113, 1.03
No. of reflections	3438
No. of parameters	436
H-atom treatment	H atoms treated by a mixture of independent and constrained refinement
Δρ_max_, Δρ_min_ (e Å^−3^)	0.19, −0.21

Computer programs: *APEX2* and *SAINT* (Bruker, 2008 ▸) and *SHELXTL* (Sheldrick, 2008 ▸).

## supplementary crystallographic information

### Crystal data

**Table:**

C_38_H_50_N_2_O_7_	*F*(000) = 1392
*M**_r_* = 646.80	*D*_x_ = 1.244 Mg m^−^^3^
Orthorhombic, *P*2_1_2_1_2_1_	Mo *K*α radiation, λ = 0.71073 Å
Hall symbol: P 2ac 2ab	Cell parameters from 2257 reflections
*a* = 10.993 (8) Å	θ = 2.4–20.4°
*b* = 13.198 (10) Å	µ = 0.09 mm^−^^1^
*c* = 23.799 (19) Å	*T* = 150 K
*V* = 3453 (5) Å^3^	Needle, colourless
*Z* = 4	0.50 × 0.10 × 0.04 mm

### Data collection

**Table:**

Bruker SMART APEXII diffractometer	3438 independent reflections
Radiation source: fine-focus sealed tube	1990 reflections with *I* > 2σ(*I*)
Graphite monochromator	*R*_int_ = 0.169
ω scans	θ_max_ = 25.1°, θ_min_ = 2.3°
Absorption correction: multi-scan (SADABS; Bruker, 2008)	*h* = −13→13
*T*_min_ = 0.959, *T*_max_ = 0.997	*k* = −15→15
19713 measured reflections	*l* = −27→28

### Refinement

**Table:**

Refinement on *F*^2^	Secondary atom site location: difference Fourier map
Least-squares matrix: full	Hydrogen site location: mixed
*R*[*F*^2^ > 2σ(*F*^2^)] = 0.060	H atoms treated by a mixture of independent and constrained refinement
*wR*(*F*^2^) = 0.113	*w* = 1/[σ^2^(*F*_o_^2^) + (0.0294*P*)^2^] where *P* = (*F*_o_^2^ + 2*F*_c_^2^)/3
*S* = 1.03	(Δ/σ)_max_ < 0.001
3438 reflections	Δρ_max_ = 0.19 e Å^−^^3^
436 parameters	Δρ_min_ = −0.21 e Å^−^^3^
0 restraints	Extinction correction: SHELXTL (Sheldrick, 2008), Fc^*^=kFc[1+0.001xFc^2^λ^3^/sin(2θ)]^-1/4^
Primary atom site location: structure-invariant direct methods	Extinction coefficient: 0.0035 (7)

### Special details

**Table:**

Geometry. All esds (except the esd in the dihedral angle between two l.s. planes) are estimated using the full covariance matrix. The cell esds are taken into account individually in the estimation of esds in distances, angles and torsion angles; correlations between esds in cell parameters are only used when they are defined by crystal symmetry. An approximate (isotropic) treatment of cell esds is used for estimating esds involving l.s. planes.
Refinement. Refinement of F^2^ against ALL reflections. The weighted R-factor wR and goodness of fit S are based on F^2^, conventional R-factors R are based on F, with F set to zero for negative F^2^. The threshold expression of F^2^ > 2sigma(F^2^) is used only for calculating R-factors(gt) etc. and is not relevant to the choice of reflections for refinement. R-factors based on F^2^ are statistically about twice as large as those based on F, and R- factors based on ALL data will be even larger.

### Fractional atomic coordinates and isotropic or equivalent isotropic displacement parameters (Å^2^)

**Table:**

	*x*	*y*	*z*	*U*_iso_*/*U*_eq_	
N1	0.3110 (4)	0.8167 (3)	0.43708 (16)	0.0312 (10)	
N2	0.4319 (4)	1.1224 (3)	0.4206 (2)	0.0436 (12)	
H11	0.407 (6)	1.116 (5)	0.457 (3)	0.10 (3)*	
O1	0.1121 (3)	0.5592 (3)	0.37321 (14)	0.0422 (9)	
O2	0.0621 (4)	0.6760 (3)	0.30922 (16)	0.0501 (11)	
O3	0.2308 (3)	0.7761 (3)	0.58012 (14)	0.0464 (10)	
O4	0.3899 (3)	0.7031 (3)	0.53704 (15)	0.0442 (9)	
O5	0.4036 (3)	0.9273 (2)	0.49501 (14)	0.0403 (9)	
O6	0.7399 (3)	1.1224 (3)	0.47147 (16)	0.0510 (10)	
O7	0.5675 (3)	1.1762 (3)	0.51165 (17)	0.0573 (12)	
C1	0.2786 (4)	0.7736 (4)	0.3819 (2)	0.0334 (12)	
H1	0.2707	0.8293	0.3537	0.040*	
C2	0.1512 (4)	0.7294 (4)	0.3953 (2)	0.0349 (13)	
H2	0.0931	0.7877	0.3943	0.042*	
C3	0.1600 (4)	0.6968 (4)	0.4560 (2)	0.0358 (13)	
H3A	0.2042	0.6318	0.4596	0.043*	
H3B	0.0784	0.6897	0.4731	0.043*	
C4	0.2309 (4)	0.7833 (4)	0.48281 (19)	0.0341 (13)	
H4	0.1733	0.8393	0.4923	0.041*	
C5	0.3734 (4)	0.6966 (4)	0.3617 (2)	0.0325 (12)	
C6	0.3970 (5)	0.6843 (4)	0.3047 (2)	0.0378 (13)	
C7	0.4838 (5)	0.6134 (4)	0.2886 (2)	0.0454 (15)	
H7	0.5010	0.6046	0.2498	0.054*	
C8	0.5454 (5)	0.5555 (5)	0.3274 (2)	0.0482 (15)	
H8	0.6047	0.5078	0.3154	0.058*	
C9	0.5203 (5)	0.5676 (4)	0.3839 (2)	0.0469 (15)	
H9	0.5608	0.5272	0.4111	0.056*	
C10	0.4355 (4)	0.6392 (4)	0.4004 (2)	0.0393 (13)	
H10	0.4199	0.6488	0.4393	0.047*	
C11	0.1050 (5)	0.6535 (4)	0.3537 (2)	0.0347 (13)	
C12	0.2549 (5)	0.4131 (4)	0.3088 (2)	0.0519 (16)	
H12	0.2946	0.4681	0.3312	0.062*	
C13	0.3418 (6)	0.3231 (5)	0.3088 (3)	0.074 (2)	
H13A	0.3423	0.2917	0.3461	0.111*	
H13B	0.4240	0.3464	0.2995	0.111*	
H13C	0.3150	0.2734	0.2809	0.111*	
C14	0.2384 (6)	0.4538 (5)	0.2488 (2)	0.0645 (18)	
H14A	0.3182	0.4697	0.2327	0.097*	
H14B	0.1886	0.5154	0.2498	0.097*	
H14C	0.1981	0.4024	0.2257	0.097*	
C15	−0.2423 (5)	0.3419 (4)	0.3906 (3)	0.0611 (18)	
H15A	−0.2895	0.2858	0.3748	0.092*	
H15B	−0.2943	0.4019	0.3940	0.092*	
H15C	−0.2118	0.3227	0.4278	0.092*	
C16	0.2962 (5)	0.7515 (4)	0.5349 (2)	0.0357 (13)	
C17	0.2759 (5)	0.7423 (5)	0.6340 (2)	0.0579 (17)	
H17A	0.2106	0.7463	0.6619	0.087*	
H17B	0.3437	0.7857	0.6457	0.087*	
H17C	0.3041	0.6721	0.6310	0.087*	
C18	0.3889 (5)	0.8945 (4)	0.4471 (2)	0.0334 (12)	
C19	0.3782 (4)	1.0456 (4)	0.3845 (2)	0.0376 (13)	
H19	0.3982	1.0647	0.3449	0.045*	
C20	0.4485 (4)	0.9445 (4)	0.3976 (2)	0.0378 (13)	
H20	0.4506	0.8986	0.3642	0.045*	
C21	0.5763 (4)	0.9835 (4)	0.4127 (2)	0.0437 (15)	
H21A	0.6346	0.9690	0.3820	0.052*	
H21B	0.6060	0.9513	0.4476	0.052*	
C22	0.5611 (5)	1.0986 (4)	0.4208 (2)	0.0412 (14)	
H22	0.5995	1.1335	0.3880	0.049*	
C23	0.6197 (5)	1.1381 (4)	0.4736 (3)	0.0430 (14)	
C24	0.8099 (5)	1.1593 (5)	0.5196 (3)	0.0618 (18)	
H24A	0.8920	1.1779	0.5073	0.093*	
H24B	0.8152	1.1059	0.5481	0.093*	
H24C	0.7695	1.2188	0.5356	0.093*	
C25	0.2412 (4)	1.0401 (3)	0.3886 (2)	0.0349 (13)	
C26	0.1694 (5)	1.0283 (4)	0.3403 (2)	0.0381 (13)	
C27	0.0444 (5)	1.0164 (4)	0.3473 (3)	0.0551 (17)	
H27	−0.0051	1.0071	0.3150	0.066*	
C28	−0.0096 (6)	1.0178 (4)	0.3992 (4)	0.066 (2)	
H28	−0.0951	1.0091	0.4025	0.080*	
C29	0.0595 (6)	1.0314 (5)	0.4460 (3)	0.0619 (19)	
H29	0.0223	1.0322	0.4821	0.074*	
C30	0.1839 (5)	1.0443 (4)	0.4410 (2)	0.0478 (15)	
H30	0.2313	1.0562	0.4738	0.057*	
C31	0.2218 (5)	1.0293 (4)	0.2826 (2)	0.0540 (17)	
H31A	0.1569	1.0182	0.2551	0.081*	
H31B	0.2827	0.9754	0.2792	0.081*	
H31C	0.2604	1.0951	0.2755	0.081*	
C32	0.3347 (5)	0.7466 (4)	0.2601 (2)	0.0518 (16)	
H32A	0.2468	0.7472	0.2671	0.078*	
H32B	0.3509	0.7170	0.2231	0.078*	
H32C	0.3659	0.8161	0.2612	0.078*	
C44	0.0484 (5)	0.4778 (4)	0.3427 (2)	0.0407 (14)	
H44A	0.0258	0.5020	0.3043	0.049*	
C45	0.1339 (5)	0.3885 (4)	0.3380 (2)	0.0451 (14)	
H45	0.1538	0.3664	0.3771	0.054*	
C46	0.0616 (5)	0.3019 (4)	0.3104 (2)	0.0557 (17)	
H46A	0.0371	0.3220	0.2720	0.067*	
H46B	0.1137	0.2409	0.3075	0.067*	
C47	−0.0515 (5)	0.2770 (4)	0.3450 (3)	0.0550 (16)	
H47A	−0.0964	0.2213	0.3264	0.066*	
H47B	−0.0260	0.2528	0.3825	0.066*	
C48	−0.1351 (5)	0.3657 (4)	0.3520 (2)	0.0468 (15)	
H48	−0.1676	0.3849	0.3142	0.056*	
C49	−0.0645 (5)	0.4561 (4)	0.3758 (2)	0.0426 (14)	
H49C	−0.1175	0.5168	0.3755	0.051*	
H49A	−0.0421	0.4418	0.4153	0.051*	

### Atomic displacement parameters (Å^2^)

**Table:**

	*U*^11^	*U*^22^	*U*^33^	*U*^12^	*U*^13^	*U*^23^
N1	0.032 (2)	0.026 (3)	0.035 (2)	−0.003 (2)	0.002 (2)	0.0005 (19)
N2	0.036 (3)	0.038 (3)	0.056 (3)	−0.003 (2)	−0.008 (3)	−0.002 (3)
O1	0.050 (2)	0.028 (2)	0.048 (2)	−0.0019 (18)	−0.012 (2)	−0.0016 (17)
O2	0.064 (3)	0.035 (2)	0.051 (2)	−0.006 (2)	−0.018 (2)	0.0069 (19)
O3	0.044 (2)	0.060 (3)	0.036 (2)	0.0077 (19)	0.006 (2)	0.0026 (18)
O4	0.042 (2)	0.040 (2)	0.050 (2)	0.0087 (19)	−0.005 (2)	0.0033 (17)
O5	0.047 (2)	0.033 (2)	0.041 (2)	−0.0036 (17)	0.002 (2)	−0.0061 (17)
O6	0.044 (2)	0.051 (3)	0.058 (3)	−0.0057 (19)	−0.005 (2)	−0.011 (2)
O7	0.045 (3)	0.068 (3)	0.059 (3)	−0.001 (2)	0.002 (2)	−0.018 (2)
C1	0.032 (3)	0.033 (3)	0.036 (3)	−0.006 (2)	0.001 (3)	−0.002 (2)
C2	0.031 (3)	0.035 (3)	0.039 (3)	0.004 (2)	−0.001 (3)	0.000 (3)
C3	0.029 (3)	0.036 (3)	0.043 (3)	−0.005 (2)	0.002 (3)	0.001 (3)
C4	0.026 (3)	0.036 (3)	0.040 (3)	0.002 (2)	0.001 (3)	−0.002 (2)
C5	0.031 (3)	0.030 (3)	0.036 (3)	−0.005 (2)	0.001 (3)	0.000 (2)
C6	0.034 (3)	0.041 (4)	0.039 (3)	−0.007 (3)	0.000 (3)	0.001 (3)
C7	0.040 (3)	0.054 (4)	0.042 (3)	0.004 (3)	0.009 (3)	−0.004 (3)
C8	0.037 (3)	0.053 (4)	0.055 (4)	0.009 (3)	0.001 (3)	−0.010 (3)
C9	0.039 (3)	0.047 (4)	0.055 (4)	0.007 (3)	−0.003 (3)	−0.001 (3)
C10	0.028 (3)	0.044 (4)	0.045 (3)	0.002 (3)	−0.004 (3)	−0.006 (3)
C11	0.024 (3)	0.031 (4)	0.049 (3)	0.003 (2)	0.003 (3)	0.006 (3)
C12	0.055 (4)	0.049 (4)	0.052 (4)	0.004 (3)	0.005 (3)	−0.014 (3)
C13	0.069 (5)	0.056 (5)	0.097 (5)	0.013 (4)	0.018 (4)	−0.013 (4)
C14	0.063 (4)	0.079 (5)	0.052 (4)	−0.006 (4)	0.009 (4)	−0.013 (3)
C15	0.062 (4)	0.045 (4)	0.076 (4)	−0.012 (3)	−0.008 (4)	0.008 (3)
C16	0.033 (3)	0.034 (3)	0.041 (3)	−0.004 (3)	0.003 (3)	0.006 (3)
C17	0.061 (4)	0.079 (5)	0.034 (3)	0.007 (3)	0.000 (3)	0.005 (3)
C18	0.031 (3)	0.029 (3)	0.040 (3)	0.004 (3)	0.001 (3)	−0.001 (3)
C19	0.038 (3)	0.036 (4)	0.039 (3)	0.001 (3)	−0.001 (3)	0.002 (3)
C20	0.034 (3)	0.033 (3)	0.047 (3)	−0.002 (2)	0.009 (3)	−0.010 (3)
C21	0.035 (3)	0.040 (4)	0.056 (4)	−0.010 (2)	0.006 (3)	−0.008 (3)
C22	0.045 (3)	0.031 (4)	0.047 (3)	−0.014 (3)	0.001 (3)	0.001 (3)
C23	0.036 (3)	0.036 (4)	0.057 (4)	−0.004 (3)	0.002 (3)	−0.002 (3)
C24	0.043 (4)	0.076 (5)	0.066 (4)	0.000 (3)	−0.007 (4)	−0.019 (3)
C25	0.035 (3)	0.022 (3)	0.048 (3)	0.002 (2)	0.004 (3)	0.003 (2)
C26	0.031 (3)	0.028 (3)	0.055 (4)	−0.001 (2)	−0.001 (3)	0.001 (3)
C27	0.040 (4)	0.042 (4)	0.084 (5)	0.001 (3)	−0.002 (4)	−0.002 (3)
C28	0.033 (4)	0.042 (4)	0.124 (7)	0.007 (3)	0.015 (5)	0.019 (4)
C29	0.048 (4)	0.055 (5)	0.083 (5)	0.021 (3)	0.018 (4)	0.019 (4)
C30	0.047 (4)	0.040 (4)	0.056 (4)	0.012 (3)	0.007 (3)	0.010 (3)
C31	0.050 (4)	0.052 (4)	0.060 (4)	0.005 (3)	−0.011 (3)	−0.016 (3)
C32	0.054 (4)	0.053 (4)	0.048 (3)	0.009 (3)	0.009 (3)	0.002 (3)
C44	0.049 (4)	0.028 (3)	0.044 (3)	−0.004 (3)	−0.011 (3)	−0.005 (3)
C45	0.044 (3)	0.040 (4)	0.051 (3)	0.003 (3)	−0.004 (3)	−0.007 (3)
C46	0.063 (4)	0.038 (4)	0.066 (4)	−0.002 (3)	0.000 (4)	−0.013 (3)
C47	0.066 (4)	0.034 (4)	0.065 (4)	−0.011 (3)	−0.015 (4)	−0.004 (3)
C48	0.044 (4)	0.038 (4)	0.058 (4)	−0.003 (3)	−0.010 (3)	0.010 (3)
C49	0.048 (3)	0.030 (3)	0.050 (3)	0.002 (3)	−0.006 (3)	0.005 (3)

### Geometric parameters (Å, º)

**Table:**

N1—C18	1.357 (6)	C15—H15C	0.9800
N1—C4	1.468 (6)	C17—H17A	0.9800
N1—C1	1.475 (6)	C17—H17B	0.9800
N2—C19	1.453 (6)	C17—H17C	0.9800
N2—C22	1.455 (7)	C18—C20	1.501 (7)
N2—H11	0.91 (6)	C19—C25	1.511 (7)
O1—C11	1.331 (6)	C19—C20	1.573 (7)
O1—C44	1.473 (6)	C19—H19	1.0000
O2—C11	1.196 (6)	C20—C21	1.538 (7)
O3—C16	1.333 (6)	C20—H20	1.0000
O3—C17	1.446 (6)	C21—C22	1.540 (7)
O4—C16	1.213 (6)	C21—H21A	0.9900
O5—C18	1.231 (5)	C21—H21B	0.9900
O6—C23	1.339 (6)	C22—C23	1.505 (7)
O6—C24	1.464 (6)	C22—H22	1.0000
O7—C23	1.185 (6)	C24—H24A	0.9800
C1—C5	1.533 (7)	C24—H24B	0.9800
C1—C2	1.550 (6)	C24—H24C	0.9800
C1—H1	1.0000	C25—C30	1.399 (7)
C2—C11	1.497 (7)	C25—C26	1.403 (7)
C2—C3	1.511 (7)	C26—C27	1.393 (7)
C2—H2	1.0000	C26—C31	1.489 (8)
C3—C4	1.523 (7)	C27—C28	1.370 (9)
C3—H3A	0.9900	C27—H27	0.9500
C3—H3B	0.9900	C28—C29	1.360 (9)
C4—C16	1.493 (7)	C28—H28	0.9500
C4—H4	1.0000	C29—C30	1.384 (8)
C5—C10	1.376 (7)	C29—H29	0.9500
C5—C6	1.389 (6)	C30—H30	0.9500
C6—C7	1.391 (7)	C31—H31A	0.9800
C6—C32	1.507 (7)	C31—H31B	0.9800
C7—C8	1.376 (7)	C31—H31C	0.9800
C7—H7	0.9500	C32—H32A	0.9800
C8—C9	1.382 (7)	C32—H32B	0.9800
C8—H8	0.9500	C32—H32C	0.9800
C9—C10	1.384 (7)	C44—C49	1.498 (7)
C9—H9	0.9500	C44—C45	1.512 (7)
C10—H10	0.9500	C44—H44A	1.0000
C12—C13	1.524 (8)	C45—C46	1.539 (7)
C12—C45	1.536 (7)	C45—H45	1.0000
C12—C14	1.537 (7)	C46—C47	1.525 (7)
C12—H12	1.0000	C46—H46A	0.9900
C13—H13A	0.9800	C46—H46B	0.9900
C13—H13B	0.9800	C47—C48	1.498 (8)
C13—H13C	0.9800	C47—H47A	0.9900
C14—H14A	0.9800	C47—H47B	0.9900
C14—H14B	0.9800	C48—C49	1.532 (7)
C14—H14C	0.9800	C48—H48	1.0000
C15—C48	1.527 (7)	C49—H49C	0.9900
C15—H15A	0.9800	C49—H49A	0.9900
C15—H15B	0.9800		
			
C18—N1—C4	118.4 (4)	C20—C19—H19	107.0
C18—N1—C1	126.9 (4)	C18—C20—C21	111.3 (5)
C4—N1—C1	113.5 (4)	C18—C20—C19	108.3 (4)
C19—N2—C22	104.4 (4)	C21—C20—C19	102.2 (4)
C19—N2—H11	112 (4)	C18—C20—H20	111.6
C22—N2—H11	106 (4)	C21—C20—H20	111.6
C11—O1—C44	118.8 (4)	C19—C20—H20	111.6
C16—O3—C17	117.1 (4)	C20—C21—C22	105.1 (4)
C23—O6—C24	116.0 (5)	C20—C21—H21A	110.7
N1—C1—C5	111.8 (4)	C22—C21—H21A	110.7
N1—C1—C2	100.4 (4)	C20—C21—H21B	110.7
C5—C1—C2	115.4 (4)	C22—C21—H21B	110.7
N1—C1—H1	109.6	H21A—C21—H21B	108.8
C5—C1—H1	109.6	N2—C22—C23	110.2 (5)
C2—C1—H1	109.6	N2—C22—C21	108.5 (4)
C11—C2—C3	117.6 (4)	C23—C22—C21	113.6 (5)
C11—C2—C1	115.0 (4)	N2—C22—H22	108.1
C3—C2—C1	104.2 (4)	C23—C22—H22	108.1
C11—C2—H2	106.4	C21—C22—H22	108.1
C3—C2—H2	106.4	O7—C23—O6	124.9 (5)
C1—C2—H2	106.4	O7—C23—C22	125.3 (5)
C2—C3—C4	102.7 (4)	O6—C23—C22	109.7 (5)
C2—C3—H3A	111.2	O6—C24—H24A	109.5
C4—C3—H3A	111.2	O6—C24—H24B	109.5
C2—C3—H3B	111.2	H24A—C24—H24B	109.5
C4—C3—H3B	111.2	O6—C24—H24C	109.5
H3A—C3—H3B	109.1	H24A—C24—H24C	109.5
N1—C4—C16	114.4 (4)	H24B—C24—H24C	109.5
N1—C4—C3	102.8 (4)	C30—C25—C26	118.8 (5)
C16—C4—C3	112.6 (4)	C30—C25—C19	120.3 (5)
N1—C4—H4	109.0	C26—C25—C19	120.9 (5)
C16—C4—H4	109.0	C27—C26—C25	118.0 (6)
C3—C4—H4	109.0	C27—C26—C31	119.6 (6)
C10—C5—C6	119.8 (5)	C25—C26—C31	122.5 (5)
C10—C5—C1	119.4 (4)	C28—C27—C26	122.3 (6)
C6—C5—C1	120.7 (5)	C28—C27—H27	118.9
C5—C6—C7	118.4 (5)	C26—C27—H27	118.9
C5—C6—C32	122.6 (5)	C29—C28—C27	119.9 (6)
C7—C6—C32	119.0 (5)	C29—C28—H28	120.0
C8—C7—C6	121.8 (5)	C27—C28—H28	120.0
C8—C7—H7	119.1	C28—C29—C30	119.8 (7)
C6—C7—H7	119.1	C28—C29—H29	120.1
C7—C8—C9	119.4 (5)	C30—C29—H29	120.1
C7—C8—H8	120.3	C29—C30—C25	121.1 (6)
C9—C8—H8	120.3	C29—C30—H30	119.4
C8—C9—C10	119.3 (5)	C25—C30—H30	119.4
C8—C9—H9	120.3	C26—C31—H31A	109.5
C10—C9—H9	120.3	C26—C31—H31B	109.5
C5—C10—C9	121.3 (5)	H31A—C31—H31B	109.5
C5—C10—H10	119.4	C26—C31—H31C	109.5
C9—C10—H10	119.4	H31A—C31—H31C	109.5
O2—C11—O1	124.3 (5)	H31B—C31—H31C	109.5
O2—C11—C2	123.6 (5)	C6—C32—H32A	109.5
O1—C11—C2	112.0 (5)	C6—C32—H32B	109.5
C13—C12—C45	112.2 (5)	H32A—C32—H32B	109.5
C13—C12—C14	110.3 (5)	C6—C32—H32C	109.5
C45—C12—C14	113.1 (5)	H32A—C32—H32C	109.5
C13—C12—H12	107.0	H32B—C32—H32C	109.5
C45—C12—H12	107.0	O1—C44—C49	105.9 (4)
C14—C12—H12	107.0	O1—C44—C45	108.1 (4)
C12—C13—H13A	109.5	C49—C44—C45	113.9 (4)
C12—C13—H13B	109.5	O1—C44—H44A	109.6
H13A—C13—H13B	109.5	C49—C44—H44A	109.6
C12—C13—H13C	109.5	C45—C44—H44A	109.6
H13A—C13—H13C	109.5	C44—C45—C12	114.0 (5)
H13B—C13—H13C	109.5	C44—C45—C46	106.9 (4)
C12—C14—H14A	109.5	C12—C45—C46	114.3 (5)
C12—C14—H14B	109.5	C44—C45—H45	107.1
H14A—C14—H14B	109.5	C12—C45—H45	107.1
C12—C14—H14C	109.5	C46—C45—H45	107.1
H14A—C14—H14C	109.5	C47—C46—C45	110.5 (5)
H14B—C14—H14C	109.5	C47—C46—H46A	109.5
C48—C15—H15A	109.5	C45—C46—H46A	109.5
C48—C15—H15B	109.5	C47—C46—H46B	109.5
H15A—C15—H15B	109.5	C45—C46—H46B	109.5
C48—C15—H15C	109.5	H46A—C46—H46B	108.1
H15A—C15—H15C	109.5	C48—C47—C46	113.1 (5)
H15B—C15—H15C	109.5	C48—C47—H47A	109.0
O4—C16—O3	123.5 (5)	C46—C47—H47A	109.0
O4—C16—C4	126.2 (5)	C48—C47—H47B	109.0
O3—C16—C4	110.1 (4)	C46—C47—H47B	109.0
O3—C17—H17A	109.5	H47A—C47—H47B	107.8
O3—C17—H17B	109.5	C47—C48—C15	112.3 (5)
H17A—C17—H17B	109.5	C47—C48—C49	109.8 (4)
O3—C17—H17C	109.5	C15—C48—C49	109.2 (5)
H17A—C17—H17C	109.5	C47—C48—H48	108.5
H17B—C17—H17C	109.5	C15—C48—H48	108.5
O5—C18—N1	120.7 (5)	C49—C48—H48	108.5
O5—C18—C20	121.0 (5)	C44—C49—C48	112.0 (4)
N1—C18—C20	118.1 (4)	C44—C49—H49C	109.2
N2—C19—C25	113.6 (4)	C48—C49—H49C	109.2
N2—C19—C20	106.0 (4)	C44—C49—H49A	109.2
C25—C19—C20	115.9 (4)	C48—C49—H49A	109.2
N2—C19—H19	107.0	H49C—C49—H49A	107.9
C25—C19—H19	107.0		
			
C18—N1—C1—C5	82.6 (6)	N1—C18—C20—C19	100.2 (5)
C4—N1—C1—C5	−110.1 (4)	N2—C19—C20—C18	85.9 (5)
C18—N1—C1—C2	−154.5 (4)	C25—C19—C20—C18	−41.1 (6)
C4—N1—C1—C2	12.8 (5)	N2—C19—C20—C21	−31.7 (5)
N1—C1—C2—C11	−163.0 (4)	C25—C19—C20—C21	−158.6 (5)
C5—C1—C2—C11	−42.6 (6)	C18—C20—C21—C22	−102.5 (5)
N1—C1—C2—C3	−32.9 (5)	C19—C20—C21—C22	12.9 (5)
C5—C1—C2—C3	87.5 (5)	C19—N2—C22—C23	−154.8 (4)
C11—C2—C3—C4	169.7 (4)	C19—N2—C22—C21	−29.9 (6)
C1—C2—C3—C4	41.1 (5)	C20—C21—C22—N2	9.5 (6)
C18—N1—C4—C16	−57.3 (6)	C20—C21—C22—C23	132.5 (5)
C1—N1—C4—C16	134.2 (4)	C24—O6—C23—O7	−2.5 (8)
C18—N1—C4—C3	−179.7 (4)	C24—O6—C23—C22	178.5 (4)
C1—N1—C4—C3	11.9 (5)	N2—C22—C23—O7	5.4 (8)
C2—C3—C4—N1	−32.2 (5)	C21—C22—C23—O7	−116.6 (6)
C2—C3—C4—C16	−155.7 (4)	N2—C22—C23—O6	−175.6 (4)
N1—C1—C5—C10	31.8 (6)	C21—C22—C23—O6	62.4 (6)
C2—C1—C5—C10	−82.1 (6)	N2—C19—C25—C30	−43.5 (6)
N1—C1—C5—C6	−148.2 (4)	C20—C19—C25—C30	79.5 (6)
C2—C1—C5—C6	97.8 (6)	N2—C19—C25—C26	138.0 (5)
C10—C5—C6—C7	0.0 (8)	C20—C19—C25—C26	−99.0 (6)
C1—C5—C6—C7	−179.9 (5)	C30—C25—C26—C27	−3.1 (7)
C10—C5—C6—C32	−178.4 (5)	C19—C25—C26—C27	175.4 (5)
C1—C5—C6—C32	1.7 (8)	C30—C25—C26—C31	176.2 (5)
C5—C6—C7—C8	0.4 (8)	C19—C25—C26—C31	−5.3 (7)
C32—C6—C7—C8	178.8 (5)	C25—C26—C27—C28	1.2 (8)
C6—C7—C8—C9	0.3 (9)	C31—C26—C27—C28	−178.1 (5)
C7—C8—C9—C10	−1.4 (9)	C26—C27—C28—C29	0.3 (9)
C6—C5—C10—C9	−1.1 (8)	C27—C28—C29—C30	0.2 (9)
C1—C5—C10—C9	178.9 (4)	C28—C29—C30—C25	−2.2 (9)
C8—C9—C10—C5	1.8 (8)	C26—C25—C30—C29	3.6 (7)
C44—O1—C11—O2	−9.1 (7)	C19—C25—C30—C29	−174.9 (5)
C44—O1—C11—C2	167.9 (4)	C11—O1—C44—C49	−102.4 (5)
C3—C2—C11—O2	158.3 (5)	C11—O1—C44—C45	135.1 (5)
C1—C2—C11—O2	−78.4 (6)	O1—C44—C45—C12	−57.3 (6)
C3—C2—C11—O1	−18.7 (6)	C49—C44—C45—C12	−174.7 (4)
C1—C2—C11—O1	104.6 (5)	O1—C44—C45—C46	175.4 (4)
C17—O3—C16—O4	−0.1 (7)	C49—C44—C45—C46	58.0 (6)
C17—O3—C16—C4	174.5 (4)	C13—C12—C45—C44	175.8 (5)
N1—C4—C16—O4	−39.9 (7)	C14—C12—C45—C44	−58.7 (6)
C3—C4—C16—O4	76.9 (6)	C13—C12—C45—C46	−60.9 (6)
N1—C4—C16—O3	145.6 (4)	C14—C12—C45—C46	64.6 (6)
C3—C4—C16—O3	−97.5 (5)	C44—C45—C46—C47	−57.6 (6)
C4—N1—C18—O5	9.4 (7)	C12—C45—C46—C47	175.3 (5)
C1—N1—C18—O5	176.1 (5)	C45—C46—C47—C48	58.6 (6)
C4—N1—C18—C20	−165.7 (4)	C46—C47—C48—C15	−175.3 (4)
C1—N1—C18—C20	1.0 (7)	C46—C47—C48—C49	−53.6 (6)
C22—N2—C19—C25	166.6 (4)	O1—C44—C49—C48	−174.8 (4)
C22—N2—C19—C20	38.2 (5)	C45—C44—C49—C48	−56.2 (6)
O5—C18—C20—C21	36.7 (7)	C47—C48—C49—C44	51.4 (6)
N1—C18—C20—C21	−148.3 (4)	C15—C48—C49—C44	174.9 (4)
O5—C18—C20—C19	−74.8 (6)		

### Hydrogen-bond geometry (Å, º)

**Table:**

*D*—H···*A*	*D*—H	H···*A*	*D*···*A*	*D*—H···*A*
C3—H3*B*···O4^i^	0.99	2.52	3.254 (6)	131
C3—H3*B*···O5^i^	0.99	2.58	3.462 (6)	149
C49—H49*A*···O7^i^	0.99	2.63	3.511 (7)	149

Symmetry code: (i) *x*−1/2, −*y*+3/2, −*z*+1.
